# *Dendropanax morbifera* Léveille extract ameliorates cadmium-induced impairment in memory and hippocampal neurogenesis in rats

**DOI:** 10.1186/s12906-016-1435-z

**Published:** 2016-11-09

**Authors:** Woosuk Kim, Hee Sun Yim, Dae Young Yoo, Hyo Young Jung, Jong Whi Kim, Jung Hoon Choi, Yeo Sung Yoon, Dae Won Kim, In Koo Hwang

**Affiliations:** 1 Department of Anatomy and Cell Biology, College of Veterinary Medicine, and Research Institute for Veterinary Science, Seoul National University, Seoul, 08826 South Korea; 2 Department of Biochemistry and Molecular Biology, Research Institute of Oral Sciences, College of Dentistry, Kangneung-Wonju National University, Gangneung, 25457 South Korea; 3 Department of Anatomy, College of Veterinary Medicine, Kangwon National University, Chuncheon, 24341 South Korea; 4 Department of Anatomy and Cell Biology, College of Veterinary Medicine, Seoul National University, Seoul, 08826 South Korea

**Keywords:** *Dendropanax morbifera* extract, Cadmium, Neurogenesis, Memory, Acetylcholinesterase

## Abstract

**Background:**

Cadmium leads to learning and memory impairment. *Dendropanax morbifera* Léveille stem extract (DMS) reduces cadmium-induced oxidative stress in the hippocampus. We investigated the effects of DMS on cadmium-induced impairments in memory in rats.

**Methods:**

Cadmium (2 mg/kg), with or without DMS (100 mg/kg), was orally administered to 7-week-old Sprague-Dawley rats for 28 days. Galantamine (5 mg/kg), an acetylcholinesterase inhibitor, was intraperitoneally administered as a positive control. A novel-object recognition test was conducted 2 h after the final administration. Cell proliferation and neuroblast differentiation were assessed by immunohistochemistry for Ki67 and doublecortin, respectively. Acetylcholinesterase activity in the synaptosomes of the hippocampus was also measured based on the formation of 5,5′-dithio-bis-acid nitrobenzoic acid.

**Results:**

An increase in the preferential exploration time of new objects was observed in both vehicle-treated and cadmium-treated rats. In addition, DMS administration increased cell proliferation and neuroblast differentiation in the dentate gyrus of vehicle-treated and cadmium-treated rats. Acetylcholinesterase activity in the hippocampal synaptosomes was also significantly higher in the DMS-treated group than in the vehicle-treated group. The effect of DMS on cadmium-induced memory impairment and cell proliferation in the hippocampus was comparable to that of galantamine.

**Conclusions:**

These results suggest that DMS ameliorates cadmium-induced memory impairment via increase in cell proliferation, neuroblast differentiation, and acetylcholinesterase activity in the hippocampus. The consumption of DMS may reduce cadmium-induced neurotoxicity in animals or humans.

## Background

Improvements in medical technology have caused sharp increases in the population of humans aged 65 years and above, and South Korea is becoming one of the most rapidly aging societies in the world. One of the most important problems in an aging society is the decrease in quality of life caused by mild cognitive impairment or the cognitive symptoms of neuropsychiatric disorders, such as schizophrenia [1, 2]. Some therapeutic agents, including donepezil, galantamine (GAL), and memantine, improve cognitive function in Alzheimer’s disease [1, 2]. However, natural extracts or compounds have been recent targets of research because of their potential for fewer associated side effects compared to many chemical compounds.

Several lines of evidence suggest that newly generated cells in the subgranular zone of the dentate gyrus can proliferate, differentiate, and integrate into the granule cell layer, where they participate in hippocampal functions, such as learning and memory [3, 4]. Proliferating cells with active cell cycles in the subgranular zone of the dentate gyrus express Ki67 [5] and adult-born neuroblasts express doublecortin (DCX) from days 1 to 28 of cell age [6, 7]. The rate of neurogenesis in the hippocampus is associated with spatial-memory consolidation [8]. The novel-object recognition test is one of most reliable methods of measuring hippocampal-dependent memory because animals remember the familiar object and increase their explorative activity (approach frequency and time spent exploring the novel object) [9]. Adult neurogenesis is required only when the reactivation session involves novelty and this process drives the updating of stored object recognition memories. Stimulation of endogenous hippocampal neurogenesis reinforces the hippocampal network and improves learning and memory, whereas ablation of hippocampal neurogenesis by γ-ray irradiation of the dentate gyrus leads to cognitive impairment [10–13]. In particular, the inhibition of WNT signaling by dominant-negative WNT significantly reduces novel-object recognition memory [13].

*Dendropanax morbifera* Léveille is an endemic plant found in southwestern Korea. The plant has been used in traditional medicine for the treatment of skin problems, infectious diseases, headaches, and other maladies [14]. *D. morbifera* Léveille stem extract (DMS) decreases free radical damage in various organs, including the hippocampus [15, 16]. In previous studies, we demonstrated that DMS facilitates the excretion of cadmium and reduces cadmium-induced oxidative damage in the hippocampus [15].

Cadmium is one of the most harmful heavy metals because it increases the permeability of the blood-brain barrier and penetrates the brain, leading to intracellular accumulation, cellular dysfunction, and cerebral edema [16–18]. The potential protective effects of DMS on hippocampal function and neuroblast differentiation after cadmium exposure have not been examined. Therefore, in this study, we established a model of cadmium-induced memory impairment in rats and investigated the effects of DMS administration on cell proliferation and neuroblast differentiation in the rat dentate gyrus after cadmium exposure.

## Methods

### Experimental animals

Male Sprague-Dawley rats were purchased from Orient Bio Inc. (Seongnam, South Korea). Rats were housed in a conventional animal facility at 23 °C with 60 % humidity, a 12 h/12 h light/dark cycle, with *ad libitum* access to food and tap water. The handling and care of the animals conformed to guidelines established in compliance with current international laws and policies (NIH Guide for the Care and Use of Laboratory Animals, NIH Publication No. 85–23, 1985, revised 1996) and were approved by the Institutional Animal Care and Use Committee of Seoul National University (SNU-130522-1). All experiments were conducted with an effort to minimize the number of animals used and the suffering caused by the procedures used in the study.

### Preparation of DMS

Fresh *D. morbifera* Léveille was obtained from Hambakjae Farm (Jeju, Korea). The plant was authenticated by two practitioners of traditional Asian medicine and a voucher specimen was deposited in our laboratory (Deposition number: 2012–030). Stems from the plant samples (100 g) were chopped, blended, soaked in 2 L of 80 % ethanol, and refluxed thrice at 20 °C for 2 h. Insoluble materials were removed by centrifugation at 10,000 *g* for 30 min, and the resulting supernatant was concentrated and freeze-dried to yield a powder (yield: 3.3 %). Before each experiment, the dried extract was dissolved in distilled and deionized water.

### Administration of cadmium, DMS, and GAL

Cadmium chloride (CdCl_2_) was obtained from Sigma-Aldrich (St. Louis, MO, USA). Animals were divided into 5 treatment groups (*n* = 10 in each group): 1) treatment with vehicle (distilled water); 2) treatment with 100 mg/kg DMS; 3) treatment with 2 mg/kg CdCl_2_; 4) treatment with CdCl_2_ and DMS; and 5) treatment with CdCl_2_ and 5 mg/kg GAL (Sigma-Aldrich). Cadmium and DMS were dissolved in distilled water and administered to 7-week-old rats, once a day for 28 days using a gavage, while GAL was dissolved in physiological saline and administered intraperitoneally for 28 days. GAL was used as a positive control in this study, owing to its action as an acetylcholinesterase (AChE) inhibitor and its ability for improving memory and promoting neurogenesis as demonstrated previously [19–21].

### Novel-object recognition test

The test was performed as described by Foyet et al. [22]. The apparatus consisted of an open box (80 × 80 × 40 cm high) made of black acryl. The floor was covered with woodchip bedding, which was moved around between trials and testing days to prevent the build-up of odor in certain places. The objects to be discriminated were made of a solid metal, and their weight ensured that the rats could not displace them. The objects were cleaned with bleach to remove the smell of the rats.

After 25 days of vehicle, cadmium, DMS and/or GAL treatment, each rat was allowed to explore the apparatus for 2 min. On the testing day (at 28 days of treatment), 2 h following the final treatment, a session of two trials, 2 min each, was carried out. In the “sample” trial (T1), 2 identical objects were placed in opposite corners of the apparatus. A rat was placed in the apparatus and was left to explore these two identical objects. After T1, the rat was placed back in its home cage and an inter-trial interval of 1 h was given. Subsequently, the “choice” trial (T2) was performed. In T2, a new object replaced one of the objects that had been presented in T1. The rats were exposed to the two different objects: one familiar and one new. Exploration was defined as directing the nose toward the object at a distance of no more than 2 cm and/or touching the object with the nose. From these measurements, a series of variables were calculated: the total time spent exploring the 2 identical objects in T1 and time spent exploring the 2 different objects, familiar and new, in T2.

The distinction between familiar and new objects in T2 was determined by comparing the time spent exploring familiar objects with that spent exploring N. The discrimination index (DI) represents the difference in exploration time expressed as a proportion of the total time spent exploring the two objects in T2.

### Tissue processing

For histology, the animals (*n* = 5 in each group) were anesthetized by an intraperitoneal injection of 1 g/kg urethane (Sigma-Aldrich) directly after the novel-object recognition test. Rats were perfused transcardially with 0.1 M phosphate-buffered saline (PBS, pH 7.4) followed by 4 % paraformaldehyde in 0.1 M PBS. The brains were dissected out and postfixed in the same fixative for 12 h. The brain tissues were cryoprotected by infiltration with 30 % sucrose overnight. Thirty-micrometer thick brain sections were serially sectioned in the coronal plane using a cryostat (Leica, Wetzlar, Germany) and collected in six-well plates containing PBS for further processing.

### Immunohistochemistry for Ki67 and DCX

To obtain accurate data for immunohistochemistry, the free-floating sections from all animals were processed carefully under the same conditions. Tissue sections were selected between 3.00 and 4.08 mm posterior to the bregma with reference to a rat brain atlas [23] for each animal. Ten sections, 90-μm apart from each other, were sequentially treated with 0.3 % hydrogen peroxide (H_2_O_2_) in PBS for 30 min and 10 % normal horse serum in 0.05 M PBS for 30 min. They were then incubated with a rabbit anti-Ki67 antibody (1:1,000; Abcam, Cambridge, UK) or goat anti-DCX antibody (1:50; Santa Cruz Biotechnology, Santa Cruz, CA) overnight at 25 °C and subsequently treated with either a biotinylated goat anti-mouse IgG or horse anti-goat IgG, and a streptavidin-peroxidase complex (1:200, Vector, Burlingame, CA). Sections were visualized by reaction with 3,3′-diaminobenzidine tetrachloride (Sigma) in 0.1 M Tris-HCl buffer (pH 7.2) and dehydrated and mounted in Canada balsam (Kanto, Tokyo, Japan) onto gelatin-coated slides.

The Ki67-positive cells were enumerated using an image-analysis system equipped with a computer-based CCD camera and Optimas 6.5 software (CyberMetrics, Scottsdale, AZ). The Ki67-positive cells in each section of the dentate gyrus were enumerated using the software protocol (CyberMetrics). The cell-counts from all of the sections for each treatment group were averaged into a single group mean.

### Measurement of AChE activity in the hippocampus

To measure AChE activity in synaptosomes, animals in each group (*n* = 5) were anesthetized with 1 g/kg urethane (Sigma-Aldrich) after the novel-object recognition test, and the hippocampi were quickly removed from the brain and placed into labeled cryo-tubes. After a quick-freeze in liquid nitrogen for 15 min, the hippocampi were homogenized in 10 volumes of an ice-cold medium (medium I), consisting of 320 mM sucrose, 0.1 mM ethylenediaminetetraacetic acid, and 5 mM 4-(2-hydroxyethyl)-1-piperazineethanesulfonic acid, pH 7.5, in a motor driven Teflon-glass homogenizer. Synaptosomes were isolated using a discontinuous Percoll gradient, as described by Nagy and Delgado-Escueta [24]. The pellet was suspended in an isoosmotic solution, and the final protein concentration was adjusted to 0.5 mg/mL. Synaptosomes were prepared fresh daily, maintained at 4 °C throughout the procedure, and used for enzymatic assays. The AChE enzymatic assay was conducted using a modification of the spectrophotometric method of Ellman et al. [25], as described by Rocha et al. [26]. The reaction mixture (2 mL final volume) contained 100 mM K^+^-phosphate buffer, pH 7.5, and 1 mM 5,5′-dithio-bis-nitrobenzoic acid. The method is based on the formation of the yellow anion, 5,5′-dithio-bis-acid nitrobenzoic, measured by reading absorbance at 412 nm during a 2-min incubation at 25 °C. The enzyme was pre-incubated for 2 min, and the reaction was initiated by adding 0.8 mM acetylthiocholine iodide (AcSCh). All samples were run in duplicate or triplicate and enzyme activity was expressed in μmol AcSCh/h/mg of protein.

### Statistical analysis

All data are expressed as the mean ± standard error of the mean (SEM). To determine the effects of cadmium, DMS, and GAL treatments, differences between the means were analyzed using one-way analysis of variance with repeated measures and a Bonferroni’s post-hoc test. Differences were considered significant if *p* ≤ 0.05.

## Results

### Effects of cadmium with or without DMS on recognition memory

**Fig. 1:**
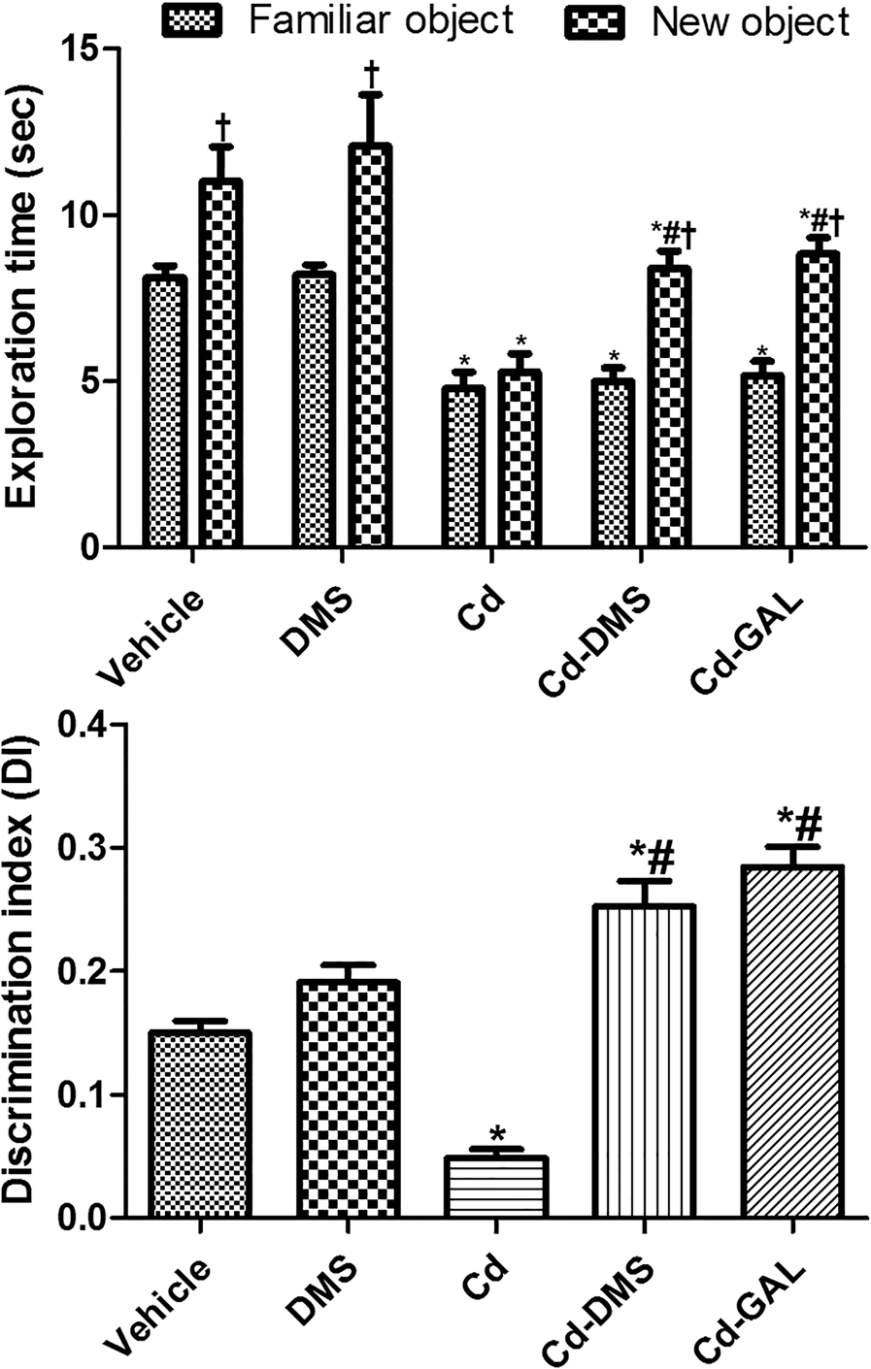
Effect of treatment on exploration time and discrimination index of familiar vs. new objects in the novel-object recognition test in rats. ^*^indicates a significant difference between the vehicle-treated and cadmium (Cd)-treated groups; ^*#*^ indicates a significant difference between the vehicle and *Dendropanax morbifera* Léveille stem extract (DMS) (or galantamine [GAL]) treatment groups; † indicates a significant difference between the familiar and new object (*p* < 0.05; *n* = 10 per group). The data are shown as mean ± SEM

During the training period, all rats showed similar behavioral patterns and spent a similar fraction of time exploring the identical objects, although the cadmium-treated group, with or without DMS, showed less activity exploring the objects compared to the control group. During the test period, rats in the control and DMS-treated group spent more time exploring new objects than familiar ones. In the cadmium-treated group, rats spent a similar amount of time exploring new and familiar objects and the DI was decreased significantly compared to that in the control group. In contrast, rats in the group treated with cadmium and DMS spent more time exploring new objects than familiar ones and the DI was increased significantly in this group compared to that of the cadmium-treated group. Rats in the group treated with cadmium and GAL also spent more time exploring new objects and the DI was increased in this group compared to that of the cadmium-treated group. In the cadmium and GAL group, the DI was similar to that in the group treated with cadmium and DMS (*F* = 17.87, df_total_ = 49, df_treatment_ = 4) (Fig. 1).

### Effects of cadmium and/or DMS on cell proliferation in the dentate gyrus

**Fig. 2:**
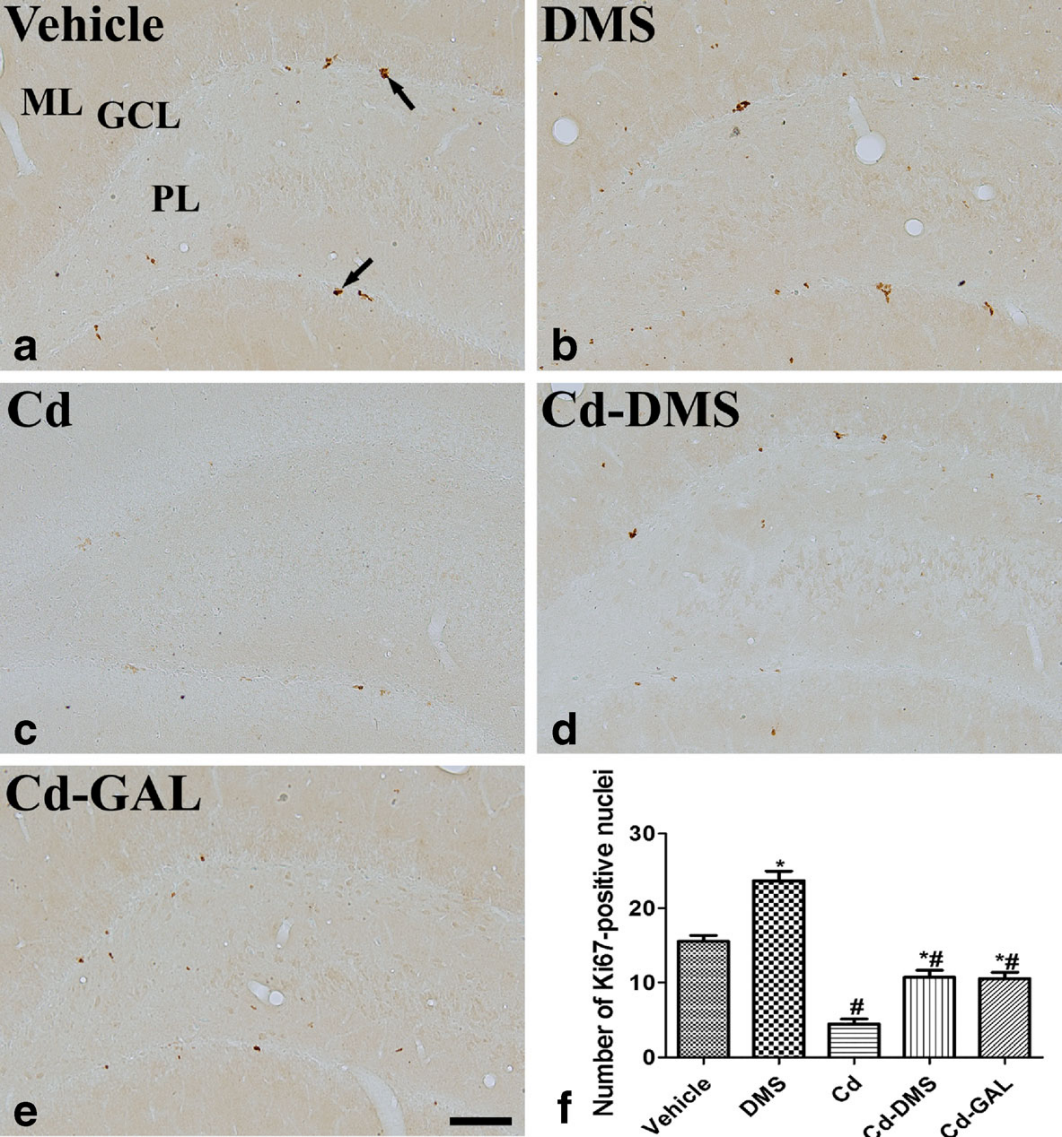
Immunohistochemistry for Ki67 in the dentate gyrus of treated rats. Ki67 immunostaining in rats treated with vehicle (**a**), *Dendropanax morbifera* Léveille stem extract (DMS; **b**), cadmium alone (Cd; **c**), cadmium and DMS (Cd-DMS; **d**), and cadmium plus galantamine (Cd-GAL) (**e**). In the vehicle-treated group, Ki67-positive nuclei (*arrows*) are detectable in the subgranular zone of the dentate gyrus. Note that there are few Ki67-positive nuclei in the Cd-treated group; however, they are comparatively abundant in the Cd-DMS-treated and Cd-GAL-treated groups. GCL, granule cell layer; ML, molecular layer; PoL, polymorphic layer. Scale bar = 100 μm. **f**: Quantitative analysis of Ki67-positive nuclei per section in the vehicle, DMS-treated, Cd-treated, DMS-Cd-treated, and DMS-GAL-treated rats using an image analyzer (*n* = 5 per group); ^*^ indicates a significant difference between the vehicle and Cd groups (*p* < 0.05); ^*#*^ indicates a significant difference between the vehicle and DMS (or GAL) groups (*p* < 0.05; *n* = 5 per group). The data are shown as mean ± SEM

In the vehicle-treated group, clustered Ki67-positive nuclei were detected in the subgranular zone of the dentate gyrus (Fig. 2a). In this group, the number of Ki67-positive nuclei was 15.6 ± 0.78 per section of the dentate gyrus. In the DMS-treated group, the number of Ki67-positive nuclei within the dentate gyrus showed a significant increase of 52.3 % compared to that in the vehicle-treated group (Fig. 2b and f). In the cadmium-treated group, few Ki67-positive nuclei were detectable in the dentate gyrus, equivalent to only 28.4 % of that found in the vehicle-treated group (Fig. 2c and f). In the group treated with cadmium and DMS, the number of Ki67-positive nuclei was increased significantly in the dentate gyrus compared to that in the cadmium-treated group, although the number was lower than that in the vehicle-treated group (Fig. 2d and f). In the group treated with cadmium and GAL, the number of Ki67-positive nuclei was also significantly increased in the dentate gyrus compared to that in the cadmium-treated group and the number of Ki67-positive nuclei was similar to the group treated with cadmium and DMS (*F* = 55.07, df_total_ = 34, df_treatment_ = 4) (Fig. 2e and f).

### Effects of cadmium and/or DMS on neuroblast differentiation in the dentate gyrus

**Fig. 3:**
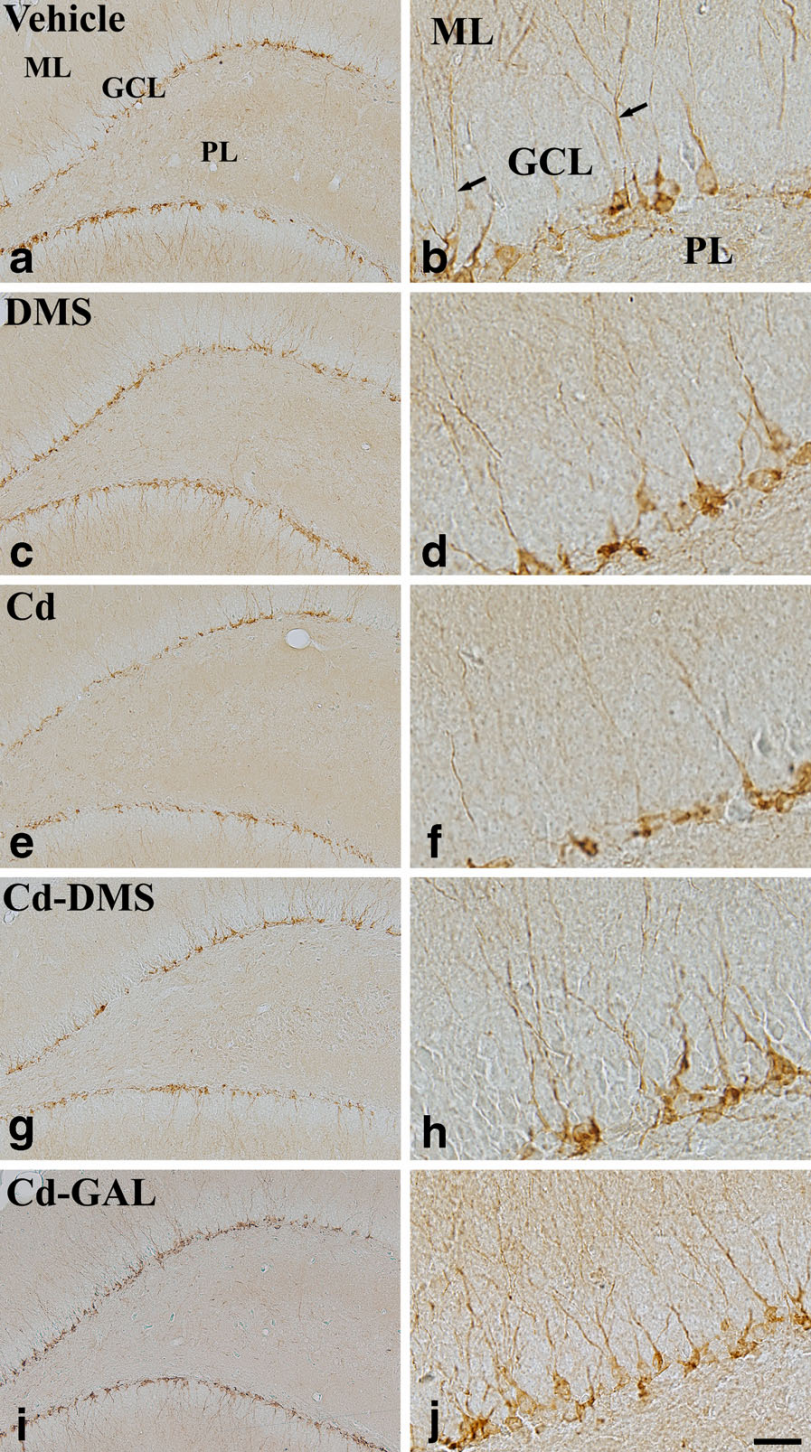
Immunohistochemistry for DCX in the dentate gyrus of treated rats. Images of doublecortin (DCX) immunostaining in rats treated with vehicle (**a**, **b**), *Dendropanax morbifera* Léveille stem extract (DMS; **c**, **d**), cadmium alone (Cd; **e**, **f**), cadmium and DMS (Cd-DMS; **g**, **h**), and cadmium and galantamine (Cd-GAL; **i**, **j**). In the vehicle-treated group, DCX-immunoreactive neuroblasts are observed in the subgranular zone with well-developed dendrites (*arrows*) extending into the molecular layer (ML). Note that there are few DCX-immunoreactive neuroblasts in the Cd-treated group, and they have poorly developed dendrites. In the Cd-DMS-treated and Cd-GAL-treated groups, there are abundant DCX-immunoreactive neuroblasts and their dendrites are well developed. GCL, granule cell layer; PoL, polymorphic layer. Scale bar = 100 μm (**a**, **c**, **e**, **g**, and **i**) and 33 μm (**b**, **d**, **f**, **h**, and **j**)

In the vehicle-treated group, DCX-immunoreactive neuroblasts were detected in the subgranular zone of the dentate gyrus, and their well-developed dendrites extended into two-thirds of the molecular layer of the dentate gyrus (Fig. 3a and b). In the DMS-treated group, the distribution pattern of neuroblasts and dendrites in the dentate gyrus were similar to those in the vehicle-treated group (Fig. 3c and d). However, in the cadmium-treated group, DCX-immunoreactive neuroblasts showed a decrease in number and a shortening of the dendrites in the dentate gyrus compared to those in the vehicle-treated group (Fig. 3e and f). In the group treated with cadmium and DMS, the DCX-immunoreactive neurons had well-developed dendrites and the number of DCX-immunoreactive neurons significantly increased compared to that in the cadmium-treated group (Fig. 3g and h). In the group treated with cadmium and GAL, DCX-immunoreactive neurons were abundantly observed in the dentate gyrus, and they also had well-developed dendrites compared to those in the cadmium-treated group (Fig. 3i and j).

### Effects of cadmium and/or DMS on AChE activity in the hippocampal homogenates

**Fig. 4:**
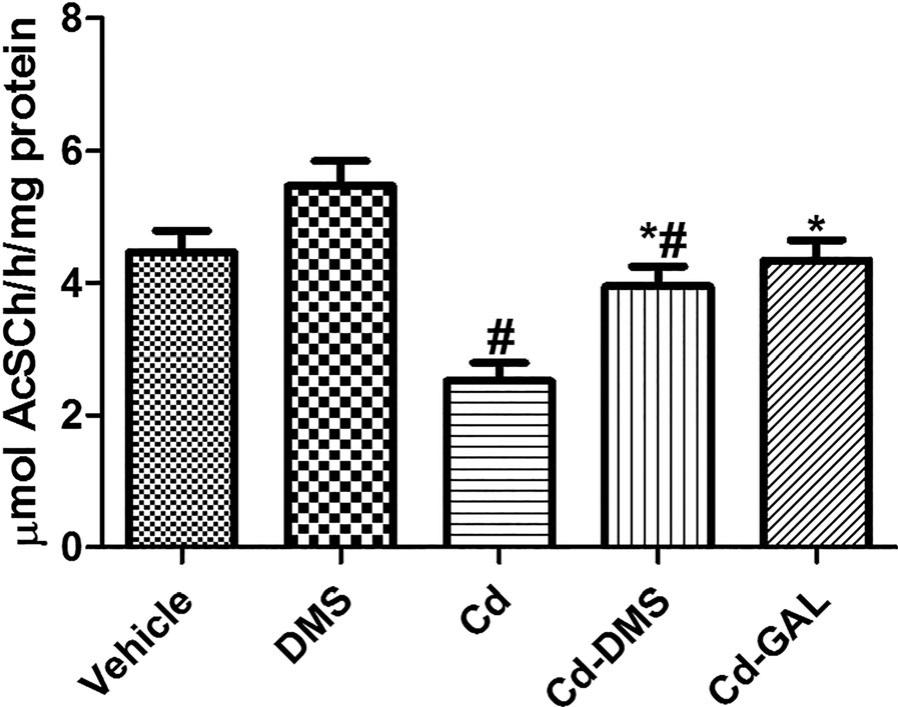
Acetylcholinesterase (AChE) activity in hippocampal synaptosomes. Graph showing hippocampal AChE activity in rats treated with vehicle, *Dendropanax morbifera* Léveille stem extract (DMS), cadmium alone (Cd), cadmium and DMS (Cd-DMS), or cadmium and galantamine (Cd-GAL). ^*^ indicates a significant difference between the vehicle and Cd groups (*p* < 0.05); ^*#*^indicates a significant difference between the vehicle and DMS (or GAL) groups (*p* < 0.05; *n* = 5 per group). The data are shown as mean ± SEM

In the vehicle-treated group, AChE activity was 4.47 μmol AcSCh/h/mg protein in the hippocampal homogenates. In the DMS-treated group, this activity was higher than that in the vehicle-treated group; however, a significant difference was not found. In the cadmium-treated group, AChE activity was decreased significantly to 56.4 % of that in vehicle-treated group. In the cadmium and DMS-treated group, as well as in the groups treated with cadmium and GAL, AChE activity showed a significant increase of 55.6 % and 72.2 %, respectively, compared to that of the cadmium-treated group (*F* = 11.36, df_total_ = 24, df_treatment_ = 4) (Fig. 4).

## Discussion

Cadmium readily accumulates in tissues (with an approximately 30-year half-life in humans), including the vascular endothelium of the brain, where it effects the integrity and permeability of the blood-brain barrier [17, 27–29]. In the present study, we observed the effects of cadmium on hippocampal function based on novel-object recognition memory. We observed that the administration of cadmium caused decreased exploratory activity, as well as a decrease in novel object recognition. This result is consistent with that of previous studies that showed that acute administration of cadmium had depressogenic effects and decreased locomotor activity [30, 31]. In addition, cadmium decreases cognitive functions in humans [32] and induces neuronal death in basal forebrain cholinergic neurons [33]. Furthermore, cadmium treatment facilitates an increase in the number and size of senile plaques and promotes memory impairments [34]. In the present study, the administration of cadmium with DMS ameliorated the decreased locomotor activity and improved the DI in a novel-object recognition test. *D. morbifera* extract is shown to have neuroprotective effects against heavy metal-induced oxidative damage [15, 35] and rotenone-induced dopaminergic cell death [36]. Extracts from the leaves and stems of *D. morbifera* decreases both cadmium-induced and mercury-induced oxidative damage in the hippocampus [15, 35]. Furthermore, rutin, isolated from *D. morbifera* Léveille, significantly decreased rotenone-induced dopaminergic cell death by ameliorating rotenone-induced c-Jun N-terminal kinase and p38 mitogen-activated protein kinase phosphorylation in SH-SY5Y cells [36].

Rapid depletion of actively dividing neurogenic cells and approximately 90 % of DCX-expressing cells by X-ray irradiation significantly decreases novel-object recognition [37]. In addition, the temporal requirement (i.e., 3 days) of neurogenesis after reactivation for reconsolidation is the same as that found for consolidation [38]. During novel-object recognition, the hippocampus is involved in the identification of spatial changes in object position and detection of novelty [39]. In the present study, we studied the effects of cadmium on the hippocampus because it is one of the brain regions most vulnerable to toxic materials. The administration of cadmium significantly reduced cell proliferation, neuroblast differentiation, and AChE activity in the hippocampus. Previous reports indicated that exposure to cadmium decreases AChE activity, increases oxidative damage in the hippocampus [18], and reduces neuronal differentiation and axonogenesis during embryonic brain development of zebrafish [40]. However, this is the first report of cadmium affecting neurogenesis in the adult brain.

Extracts from the leaves of *D. morbifera* suppress the production of pro-inflammatory cytokines and increase anti-inflammatory marker genes in lipopolysaccharide-stimulated BV2 cells [41]. In the present study, we also investigated the effects of DMS on the cadmium-induced decrease in hippocampal function. Administration of DMS significantly ameliorated cadmium-induced impairment of novel-object recognition memory. In addition, DMS increased cell proliferation, neuroblast differentiation, and AChE activity in the hippocampus, thus countering the effects of cadmium exposure. DMS had a comparable potency to GAL in terms of increasing cell proliferation, neuroblast differentiation, and AChE activity in the hippocampus. GAL has been reported to increase neurogenesis in the subgranular zone of the dentate gyrus and in the subventricular zone of the lateral ventricle [19–21]. Collectively, our results suggest that the administration of DMS significantly increases cell proliferation and neuroblast differentiation by promoting AChE activity in the dentate gyrus, where AChE is highly expressed in proliferating and differentiating cells and is likely involved in neurite outgrowth [42–44]. This conclusion is supported by previous studies that showed that AChE is involved in progenitor-cell proliferation and differentiation [45], and modulation of the cholinergic system significantly alters hippocampal neurogenesis [46, 47]. The mechanism of DMS action was not demonstrated in this study; however, DMS has possible neuroprotective effects against cadmium-induced neurotoxicity. Rutin and/or its metabolites, components arising from DMS, are able to cross the blood brain barrier [48, 49], and rutin may reduce memory deficits in Alzheimer’s disease [50].

## Conclusion

Repeated exposure to cadmium causes memory impairment and reduces cell proliferation, neuroblast differentiation, and AChE activity. The administration of DMS effectively ameliorates these cadmium-induced effects by maintaining AChE activity in the hippocampus.

## Abbreviation

AChE: Acetylcholinesterase
AcSCh: Acetylthiocholine iodide
DCX: Doublecortin
DI: Discrimination index
DMS: *D. morbifera* Léveille stem extract
GAL: Galantamine
PBS: Phosphate-buffered saline
SEM: Standard error of the mean
